# Impact of the fluid challenge infusion rate on cardiac stroke volume during major spinal neurosurgery: a prospective single center randomized interventional trial

**DOI:** 10.1186/s12871-022-01945-6

**Published:** 2022-12-23

**Authors:** Götz Schmidt, Emmanuel Schneck, Fabian Edinger, Fidaa Jablawi, Eberhard Uhl, Christian Koch, Michael Sander

**Affiliations:** 1 Department of Anesthesiology, Operative Intensive Care Medicine and Pain Therapy, Justus Liebig University of Giessen, Rudolf-Buchheim-Strasse 7, 35392 Giessen, Germany; 2 Department of Neurosurgery, University of Giessen, Justus Liebig University of Giessen, Klinikstrasse 33, 35392 Giessen, Germany

**Keywords:** Spinal surgery, Fluid therapy, Crystalloid, Colloid, Goal directed therapy

## Abstract

**Background:**

Fluid therapy, including the choice of a crystalloid or colloid infusion, the execution time of a volume bolus, and the expected volume need of a patient during surgery, varies greatly in clinical practice. Different goal directed fluid protocols have been developed, where fluid boluses guided by dynamic preload parameters are administered within a specific period.

**Objective:**

To study the efficacy of two fluid bolus infusion rates measured by the response of hemodynamic parameters.

**Design:**

Monocentric randomized controlled interventional trial.

**Setting:**

University hospital.

**Patients:**

Forty patients undergoing elective major spinal neurosurgery in prone position were enrolled, thirty-one were finally analyzed.

**Interventions:**

Patients were randomly assigned to receive 250 ml crystalloid and colloid boluses within 5 min (group 1) or 20 min (group 2) when pulse pressure variation (PPV) exceeded 14%.

**Main outcome measures:**

Changes in stroke volume (SV), mean arterial pressure (MAP), and catecholamine administration.

**Results:**

Group 1 showed a greater increase in SV (*P* = 0.031), and MAP (*P* = 0.014), while group 2 still had higher PPV (*P* = 0.005), and more often required higher dosages of noradrenalin after fluid administration (*P* = 0.033). In group 1, fluid boluses improved CI (*P* < 0.01), SV (*P* < 0.01), and MAP (*P* < 0.01), irrespective of whether crystalloids or colloids were used. In group 2, CI and SV did not change, while MAP was slightly increased (*P* = 0.011) only after colloid infusion.

**Conclusions:**

A fluid bolus within 5 min is more effective than those administered within 20 min and should therefore be the primary treatment option. Furthermore, bolus infusions administered within 20 min may result in volume overload without achieving relevant hemodynamic improvements.

**Trial registration:**

German Clinical Trials Register: DRKS00022917.

**Supplementary Information:**

The online version contains supplementary material available at 10.1186/s12871-022-01945-6.

## Background

Fluid therapy is daily routine practice in every operating theatre before, during, and after surgery. While the goal of every volume administration is preserving hemodynamic stability and intracellular homeostasis, a distinction can be made between maintenance and restoration fluid therapy [1, 2]. Perioperative maintenance fluid continuously provides daily water and electrolyte requirements, while restoration and resuscitation fluids replace blood and fluid losses to ensure sufficient cardiac preload and oxygen delivery. Fluids are often administered as bolus infusions delivering a particular volume in a limited time [2]. Balanced isotonic crystalloid solutions are the most frequent fluid type used for fluid maintenance [3]. However, the intravascular volume effect of crystalloids is low and hemodynamic resuscitation with colloids was found to be faster and required less amount of fluid in patients with clinical hypovolemia compared to the same volume of crystalloids [1, 4]. However, the choice of fluid, the execution of a volume bolus, and a patient’s expected volume need are characterized by strong local and inter-individual variability [5–7]. Therefore, goal directed fluid therapy (GDT) has been widely used in clinical practice where fluid boluses are guided by cardiac stroke volume (SV), or dynamic preload parameters such as pulse pressure variation (PPV) and stroke volume variation (SVV). Although preload-guided fluid restoration is primarily performed using consecutive steps of a fixed volume of infusion, the period in which a fluid challenge is performed largely differs among protocols, from as fast as possible to less than 5 or 10 min, or within 15 min [8–11]. However, the effect of the time within a fluid bolus is administered on the hemodynamic response relating to cardiac SV and other hemodynamic parameters remains largely unknown. Thus, we hypothesized that a higher infusion rate may trigger a stronger hemodynamic response after a distinct fluid challenge. Consequently, this randomized controlled trial aimed to evaluate the impact of two different infusion rates on cardiac SV, after fluid challenges triggered by PPV.

## Methods

### Study design

This single center randomized prospective interventional trial prospectively enrolled consecutive patients at the university hospital of Giessen, Germany. Ethical approval for this study (AZ 258/19) was provided by the local ethics committee of the medical faculty of the Justus-Liebig-University, Giessen, Germany on 13 March 2020, and this trial was registered with the German Clinical Trials Register (DRKS00022917; Date of registration: 04/09/2020). All patients gave informed consent to participate in this trial. Patients undergoing elective major spinal neurosurgery in general anesthesia and prone position were screened for inclusion criteria, including age ≥ 18 years, presence of sinus rhythm, and the routine need for invasive blood pressure monitoring. Patients were randomized immediately after providing informed consent using opaque envelopes containing the group assignment. Patients were assigned to receive fluid boluses within 5 or 20 min, respectively. Baseline data of the two study groups, including age, sex, body mass index (BMI), medical and clinical history, awake CI, and SV, were compared to ensure adequate matching. Exclusion criteria were emergency surgery, American Society of Anesthesiology (ASA) IV-VI classification, acute heart failure, chronic renal failure greater than stage II according to Kidney Disease: Improving Global Outcomes (KDIGO) criteria, arterial disease greater than stage IIb, atrial fibrillation, and pregnancy or nursing [12].

### Anesthetic management

After radial artery cannulation and zero calibration, CI, SV, and MAP were measured in supine position before anesthesia induction using the uncalibrated HemoSphere advanced monitoring platform with FloTrac IQ® transducer (Edwards Lifesciences, Irvine, CA, USA). General anesthesia was induced with propofol (1 – 2 mg kg^−1^), sufentanil (0.3 – 0.5 µg kg^−1^), and cis-atracurium (0.15 mg kg^−1^), while maintenance was achieved with sevoflurane (0.8 – 1 MAC) and repetitive boluses of sufentanil (0.1 – 0.2 µg kg^−1^) and cis-atracurium (0.03 mg kg^−1^). MAP was targeted at 65—80 mmHg using 0.5 – 1 ml boluses of cafedrine/theodrenaline 200 mg/10 mg, or continuously infused noradrenaline. All patients underwent endotracheal intubation, while establishing a central venous catheter was at the anesthesiologist’s discretion. After the patients were placed in the prone position, intraoperative pressure-controlled ventilation was set at a tidal volume of ≥ 8 ml kg^−1^ ideal body weight (IBW, estimated with body height minus 100 cm) and at respiratory rates maintaining a physiological end-tidal and arterial partial pressures of carbon dioxide. Before skin incision, all patients received less than 500 ml of crystalloid infusion (Sterofundin ISO, B. Braun, Melsungen, Germany) followed by a restrictive baseline crystalloid infusion of 4 ml kgIBW^−1^ via an infusion pump [13]. Blood transfusion and coagulation management were performed according to the current guidelines from the European Society of Anesthesiology on the management of severe perioperative bleeding [14].

### Treatment algorithm

During surgery, PPV was continuously assessed. Once it reached ≥ 14%, a 250 ml fluid bolus was given within the allocated period. Crystalloid boluses (Sterofundin, B. Braun, Melsungen, Germany) were used for the first two consecutive interventions, then any further intervention was performed with colloids (4% gelatin, Gelafundin, B. Braun, Melsungen, Germany) according to local clinical practice [15]. If PPV remained ≥ 14%, the next intervention began immediately. Once PPV dropped below 14%, no further intervention was performed until PPV was again ≥ 14%. The treatment protocol was applied between skin incision and last suture while all patients were placed in the prone position, and stable conditions of general anesthesia were ensured.

### Endpoints

The intervention-based primary endpoint of this study was the change in cardiac SV after the intervention. Further endpoints included the percentage of interventions improving SV > 10%, MAP, CI, and the administration of noradrenaline. Hemodynamic data were recorded automatically every 20 s.

### Statistical analysis

No formal power analysis was possible due to the absence of previous clinical data. A sample size of 20 patients generating approximately 50 interventions per treatment group was considered suitable for detecting clinically relevant differences in the hemodynamic parameters. Further exploratory measures included intraoperative hypotension (IOH) which was defined as MAP < 65 mmHg for ≥ 1 min. Frequency (n), absolute (min) and relative duration (min % of the incision-suture time^−1^), absolute and relative area under the curve (AUC) of IOH (mmHg × min and mmHg × min % of the incision-suture time^−1^) were calculated. Hemodynamic data were extracted from the HemoSphere advanced monitoring platform, and clinical data were obtained from the local electronic automated patient data management system (IMESO® GmbH, Giessen, Germany). Categorical variables are presented as numbers and percentages, while continuous variables are presented as median [interquartile range (IQR)]. Data were compared using the Mann–Whitney-Wilcoxon test for continuous data and the χ^2^ test with Yates’ continuity correction or Fisher’s exact test for categorical data. Results with two-tailed *P* < 0.05 were considered statistically significant. Statistical analysis was performed using IBM SPSS Statistics, version 28.0.0.1 (IBM, Armonk, NY, USA).

## Results

**Fig. 1:**
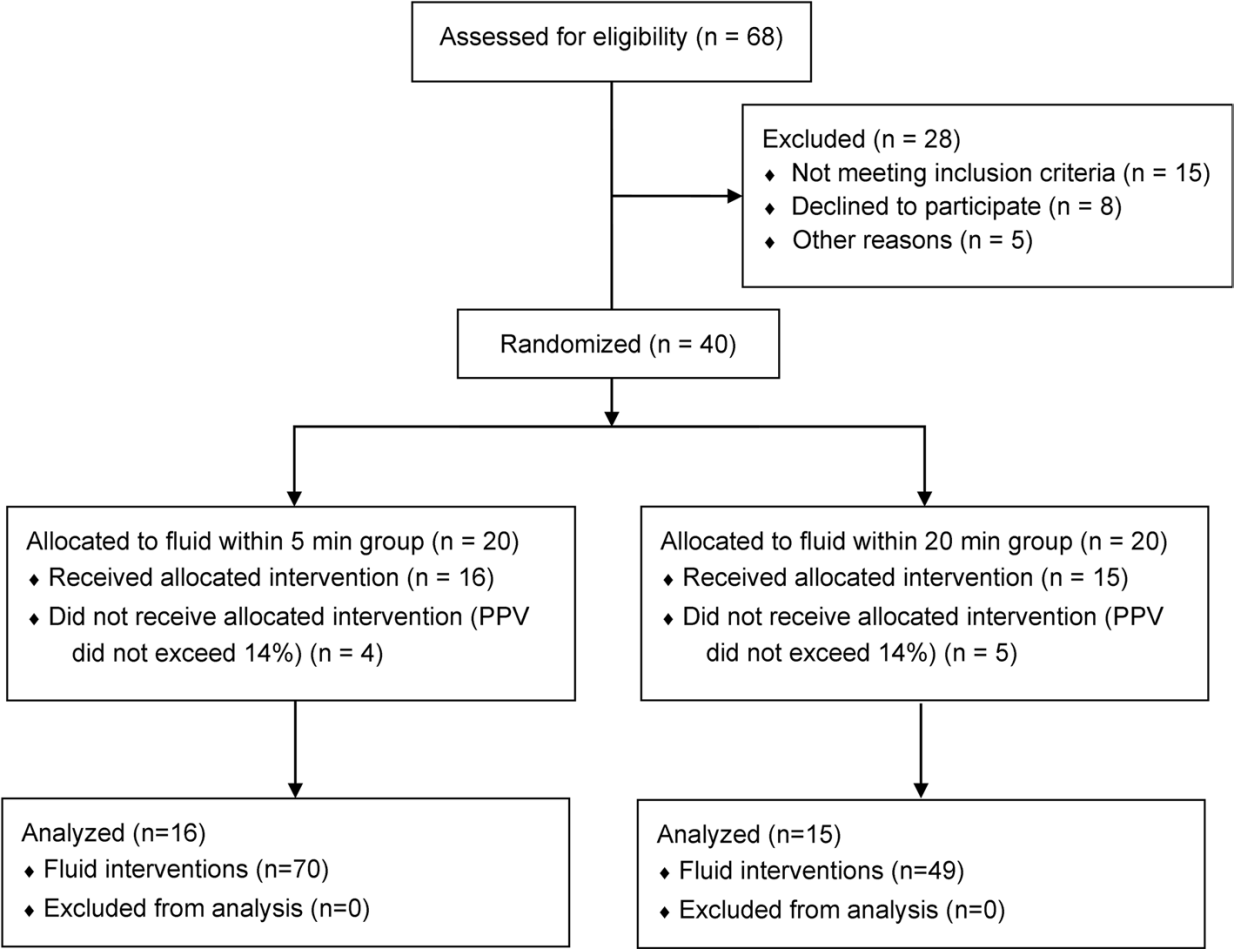
Study flowchart

Forty consecutive patients fulfilling the inclusion criteria were prospectively enrolled between August 2020 and December 2021. Patients were randomized into group 1 (bolus within 5 min, equivalent to an infusion rate of 50 ml min^−1^) or group 2 (bolus within 20 min, equivalent to an infusion rate of 12.5 ml min^−1^) in a balanced manner (Fig. 1). Four (group 1) and five (group 2) patients had to be excluded because they did not require any fluid bolus during surgery. Finally, group 1 comprised 16 patients with 70 (29 crystalloid, 41 colloid) fluid boluses, and group 2 comprised 15 patients with 49 (26 crystalloid, 23 colloid) fluid boluses.

### Patient characteristics and intraoperative characteristics

Overall characteristics are summarized in Table 1. Most patients were male (13 (81.3%) in group 1 vs. 10 (66.7%) in group 2, *P* = 0.433), while median age was 66 [57 to 77] years in group 1 and 63 [54 to 70] years in group 2 (*P* = 0.572). Patients in both groups were noted to have a high prevalence of cardiopulmonary comorbidities, which did not differ significantly between the two treatment groups. Awake hemodynamics, including CI, SV, and MAP, were also comparable. No differences were measured between the groups regarding the duration of surgery and anesthesia, and the number of administered fluid boluses. While group 1 received a higher total volume of fluids (*P* = 0.045) due to greater crystalloid infusion (*P* = 0.027), the volumes of colloids, red blood cell concentrate, and fresh frozen plasma were comparable. The cumulative amount of cafedrine/theodrenaline, and noradrenaline administered, and blood loss, urinary output, and total fluid loss did not differ significantly between groups.

**Table 1:** Overall characteristics

Characteristics	Group 1 (5 min) *n* = 16	Group 2 (20 min) *n* = 15	*P*
Patient characteristics			
Male—no. (%)	13 (81.3)	10 (66.7)	0.433
Median age [IQR]—years	66 [57—77]	63 [54—70]	0.572
Mean Body-Mass-Index [IQR] – kg m^−2^	29.9 [25.8—30.9]	26.1 [23.4—30.8]	0.318
ASA-Score			
ASA 1—no. (%)	2 (12.5)	0 (0)	0.484
ASA 2—no. (%)	5 (31.3)	3 (20.0)	0.685
ASA 3—no. (%)	9 (56.3)	12 (80)	0.252
Pre-existing conditions			
Arterial hypertension—no. (%)	12 (75.0)	10 (66.7)	0.704
Coronary artery disease—no. (%)	3 (18.8)	5 (33.3)	0.433
Prior myocardial infarction—no. (%)	2 (12.5)	1 (6.7)	1.0
Diabetes mellitus—no. (%)	4 (33.3)	3 (20.0)	1.0
Chronic pulmonary obstructive disease—no. (%)	2 (12.5)	1 (6.6)	1.0
Congestive heart failure—no. (%)	1 (6.3)	0 (0.0)	1.0
Peripheral vessel disease—no. (%)	2 (12.5)	1 (6.7)	1.0
Chronic kidney disease—no. (%)	2 (12.5)	0 (0)	0.484
Awake haemodynamics			
Cardiac index [IQR]—l/minm^2^	3.2 [2.5—3.5]	2.9 [2.6—4.0]	0.861
Stroke volume [IQR]—ml	86 [78—102]	95 [73—118]	0.626
Mean arterial pressure [IQR]—mmHg	103 [89—109]	105 [96—116]	0.861
Anaesthesia			
Duration of surgery [IQR]—min	167 [150—226]	124 [107—190]	0.101
Duration of anaesthesia [IQR]—min	298 [270—335]	244 [205—317]	0.086
Fluid boluses			
Crystalloid—no. (%)	29 (41.4)	26 (53.1)	0.286
Colloid—no (%)	41 (58.6)	23 (46.9)	0.286
Intraoperative fluid therapy			
Number of fluid boluses [IQR]—no	4 [3–6]	3 [2–4]	0.129
Total amount of fluids [IQR]—ml	2791 [1987—3108]	1664 [1330—2765]	**0.045**
Amount of crystalloids [IQR]—ml	1948 [1718—2212]	1490 [1311—1958]	**0.027**
Amount of colloids [IQR]	750 [250—1000]	250 [0—625]	0.129
Amount of red blood cell concentrate [IQR]—ml	0 [0—225]	0 [0—0]	0.861
Amount of fresh frozen plasma [IQR]—ml	0 [0 – 0]	0 [0 – 0]	1.0
Catecholamine therapy			
Cafedrine/theodrenaline [IQR]—ml	1.1 [1.0—1.9]	2.0 [1.0—2.5]	0.188
Noradrenaline [IQR]—µg	101 [0—317]	56 [0—151]	0.545
Loss of body fluids			
Total fluid loss [IQR]—ml	1165 [945—1513]	950 [470—1680]	0.682
Blood loss [IQR]—ml	550 [338—800]	300 [175—725]	0.423
Urinary output [IQR]—ml	450 [350—963]	500 [230—1060]	0.800

### Hemodynamic measurements and catecholamine requirements after bolus infusions

**Fig. 2:**
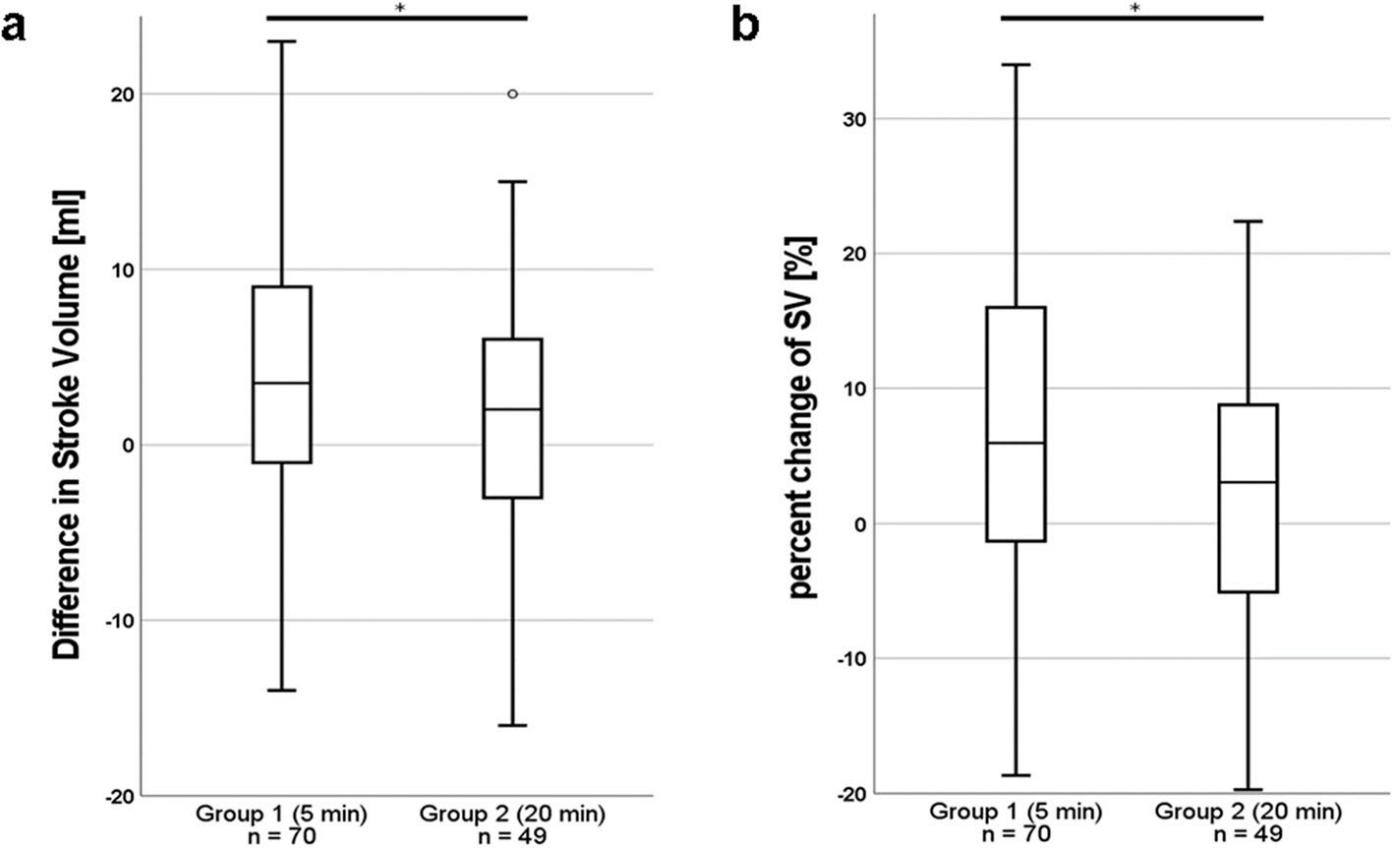
**a** Increase in cardiac stroke volume was significantly greater in group 1 (fluid challenge within 5 min) than in group 2 (fluid challenge within 20 min; 4 [-1 – 9] ml vs. 2 [-2 – 6] ml, *p = 0.031). **b** Relative increase of SV (%change of SV) in group 1: + 6.0 [-1.4—16.2]% vs. + 3.1 [-5.2—10.2]% in group 2, *p = 0.028

When a fluid bolus was indicated based on the treatment protocol, hemodynamic measurements including PPV were comparable between the two groups (Table 2). Absolute increase in SV (*p* = 0.031), and percent change of SV (*p* = 0.028) were significantly greater in group 1 than in group 2, while absolute values of SV remained comparable (Fig. 2). The increase in MAP was significantly lower in group 2 (*p* = 0.014), and furthermore, the decrease in PPV was lower in group 2 (*p* = 0.008), which subsequently resulted in an overall increased PPV after fluid challenges in group 2 (*p* = 0.005). An increase of SV greater than 10% was seen in numerically higher fractions in group 1, although not statistically significant. Noradrenaline administration remained on the same dosage more often in group 1 than in group 2 (*P* = 0.015), while in group 2, noradrenaline requirements were more frequently elevated (*P* = 0.033). Greater changes in PPV, SV and MAP and the differences in catecholamine requirements were found only after colloid boluses in group 1 (Table 3). No significant changes in ΔSV, ΔMAP, and catecholamine requirements were observed with crystalloid infusions. Hemodynamics and catecholamine requirements stratified by crystalloid and colloid infusion are shown in Supplementary Tables 1 and 2.

**Table 2:** Comparison of hemodynamics and catecholamine requirements between two infusion speeds

Characteristics	Group 1 (5 min) *n* = 70	Group 2 (20 min) *n* = 49	*P*
Pulse pressure variation			
PPV prior to bolus infusion [IQR]—%	15 [14–16]	15 [14–16]	0.697
PPV after bolus infusion [IQR]—%	10 [8–12]	12 [10–14]	**0.005**
Alteration			
ΔSV > 10%—no (%)	28 (40.0)	12 (24.5)	0.117
%change of SV [IQR]—%	6.0 [-1.4—16.2]	3.1 [-5.2—10.2]	**0.028**
%change of CI [IQR]—%	4.3 [0.0—10.0]	0.0 [-5.3—7.0]	0.129
ΔMAP [IQR]—mmHg	8 [1–14]	2 [-2—9]	**0.014**
ΔPPV [IQR]—%	-6 [-2—(-8)]	-3 [-6—(-0.5)]	**0.008**
Noradrenaline requirements			
less—no (%)	7 (10.0)	8 (16.3)	0.458
equal—no (%)	58 (82.9)	30 (61.2)	**0.015**
more—no (%)	5 (7.1)	11 (28.9)	**0.033**

*PPV* pulse pressure variation, *CI* cardiac index, *SV* stroke volume, *MAP* mean arterial pressure

**Table 3:** Comparison of hemodynamics and catecholamine requirements between two infusion speeds stratified by fluid type

**Crystalloids only**	**Group 1 (5 min)** ***n* = 29**	**Group 2 (20 min)** ***n* = 26**	***P***
Haemodynamic alterations			
ΔSV [IQR]—ml	2 [-1.5—9]	1.5 [-4—6.5]	0.340
ΔSV [IQR] > 10%—no (%)	9 (31.0)	6 (23.1)	0.720
ΔMAP [IQR]—mmHg	4 [0—10]	0 [-6—11]	0.307
ΔPPV [IQR]—%	-6 [-8—(-2)]	-3 [-6 – 0]	0.109
Noradrenaline			
less—no (%)	1 (3.4)	3 (11.5)	0.265
equal—no (%)	25 (86.2)	18 (69.2)	0.232
more—no (%)	3 (10.3)	5 (19.2)	0.291
**Colloids only**	**Group 1 (5 min)** ***n* = 41**	**Group 2 (20 min)** ***n* = 23**	***P***
Haemodynamic alterations			
ΔSV [IQR]—ml	5 [-1—9]	2 [-2—7]	**0.044**
ΔSV [IQR] > 10%—no (%)	19 (46.3)	6 (36.1)	0.185
ΔMAP [IQR]—mmHg	9 [6–15]	3 [-1—10]	**0.021**
ΔPPV [IQR]—%	-5 [-9—(-3)]	-3 [-6—(-1)]	**0.036**
Noradrenaline			
less—no (%)	6 (14.6)	5 (21.7)	0.347
equal—no (%)	33 (80.5)	12 (52.2)	**0.036**
more—no (%)	2 (4.9)	6 (26.1)	**0.021**

SV stroke volume, *MAP* mean arterial pressure

### Efficacy of certain fluid boluses

Administration of a fluid bolus in group 1 caused significant CI and MAP improvement after crystalloid and colloid infusion (*P* < 0.01; Table 4). In contrast, CI and SV remained unchanged in group 2, while MAP improved significantly after a fluid challenge (*P* = 0.011). However, MAP increased only after colloid infusion (*P* = 0.018), while CI, SV, and MAP did not change after crystalloid infusion in group 2.

**Table 4:** Efficacy of fluid boluses in improving hemodynamics stratified by fluid type and infusion speed

Characteristics	Before fluid bolus	After fluid bolus	*P*
Group 1: Crystalloid & Colloid within 5 min	*n* = 70		
Cardiac index [IQR] – l min^−1^ m-^2^	2.1 [1.7—2.6]	2.2 [1.8—2.6]	** < 0.01**
Stroke volume [IQR]—ml	64 [53—74]	70 [57—80]	** < 0.01**
Mean arterial pressure [IQR]—mmHg	74 [68—79]	82 [74—91]	** < 0.01**
Crystalloid within 5 min	*n* = 29		
Cardiac index [IQR]—l min^−1^ m-^2^	2.2 [1.7—2.6]	2.3 [1.8—2.7]	**0.039**
Stroke volume [IQR]—ml	67 [53—77]	74 [55—80]	** < 0.01**
Mean arterial pressure [IQR]—mmHg	74 [70—80]	80 [73—87]	** < 0.01**
Colloid within 5 min	*n* = 41		
Cardiac index [IQR]—l min^−1^ m-^2^	2.0 [1.6—2.7]	2.2 [1.8—2.7]	** < 0.01**
Stroke volume [IQR]—ml	62 [53—72]	70 [57—80]	** < 0.01**
Mean arterial pressure [IQR]—mmHg	73 [67—79]	88 [78—94]	** < 0.01**
Group 2: Crystalloid & Colloid within 20 min	*n* = 49		
Cardiac index [IQR]—l min^−1^ m-^2^	2.2 [1.8—2.7]	2.3 [1.8—2.7]	0.288
Stroke volume [IQR]—ml	60 [58—69]	64 [60—72]	0.229
Mean arterial pressure [IQR]—mmHg	74 [69—80]	77 [72—87]	**0.011**
Crystalloid within 20 min	*n* = 26		
Cardiac index [IQR]—l min^−1^ m-^2^	2.1 [1.8—2.7]	2.3 [1.9—2.5]	0.362
Stroke volume [IQR]—ml	61 [57—71]	63 [60—74]	0.484
Mean arterial pressure [IQR]—mmHg	75 [69—84]	78 [73—88]	0.192
Colloid within 20 min	*n* = 23		
Cardiac index [IQR]—l min^−1^ m-^2^	2.3 [1.7—2.8]	2.5 [1.8—2.8]	0.540
Stroke volume [IQR]—ml	60 [58—70]	64 [60—68]	0.261
Mean arterial pressure [IQR]—mmHg	73 [68—78]	77 [71—87]	**0.018**

### Intraoperative hypotension

Measurements of IOH incidence, absolute and relative IOH duration, and absolute and relative IOH AUC are summarized in Table 5 and did not differ significantly.

**Table 5:** Intraoperative hypotension

Characteristics	Group 1 (5 min) *n* = 16	Group 2 (20 min) *n* = 15	*P*
Intraoperative hypotension > 1 min [IQR]—no	1 [0—3]	1 [0—3]	0.711
Absolute duration of IOH [IQR]—min	1.8 [0—6.9]	8.0 [0.2—14.5]	0.470
Relative duration of IOH [IQR] – min %^−1^	1.0 [0—5.9]	5.9 [0—11.0]	0.318
Absolute AUC MAP < 65 mmHg [IQR]—mmHg × min	3.7 [0—30.1]	27.3 [0—53.3]	0.281
Relative AUC MAP < 65 mmHg [IQR]—mmHg × min %^−1^	2.3 [0—18.2]	18.3 [0—47.8]	0.216

*IOH* intraoperative hypotension, *AUC* area under the curve, *MAP* mean arterial pressure

## Discussion

Our prospective randomized trial evaluated two fluid challenge infusion rates on their efficacy concerning the initial intraoperative hemodynamic response. We have shown that a fluid bolus within 5 min had a stronger effect on the improvement of cardiac SV regardless of the infusion type used, while a fluid bolus given within 20 min had no significant influence on cardiac hemodynamic measurements. These findings should be carefully considered when evaluating different GDT strategies because the time in which a fluid challenge is given varies among GDT protocols used in several clinical trials. While the proposed fluid volumes range between 100 and 500 ml, the time within which a fluid challenge is given varies between 5 and 15 min, with most given within 10 min [2, 7, 9–11].

A distinct administered fluid bolus must sufficiently stretch cardiac sarcomeres, increasing right ventricular end diastolic volume [16]. Whether a fluid administration within a longer period can meet this requirement must be questioned since our study provides evidence that a fluid bolus within 20 min cannot fulfil these requirements. Furthermore, the intravascular volume effect of the given fluids may not have been sufficient to provoke a significant effect via the Frank-Starling law. Both redistributions in interstitial spaces and large compliant veins and stress-relaxation of the vessel wall can return intravascular filling pressures to baseline within a short period [17, 18]. Crystalloid use within 20 min appears to be particularly inappropriate since crystalloids are rapidly redistributed within minutes, and their intravascular volume effect is below 20%, leading to a subsequent decrease in SV even in initial fluid responders [4, 18]. In addition, clinicians use fluid challenges to quickly assess the potential for improvements in cardiac preload, evaluating the potential benefit of further fluid administrations. This intention may be impeded by a longer period and ineffective intravascular volumes, and by external factors such as intraoperative surgical steps changing the patient’s hemodynamics, leading to unusable results. While the time span within which a fluid bolus is given varies among GDT protocols, an increase in SV > 10% is universally accepted as indicating fluid responsiveness, supporting further volume boluses [7]. However, our findings suggest that a 250 ml fluid challenge given > 5 min underestimates fluid responsiveness based on that cut-off because a median SV increase of > 10% was barely reached in treatment group 1.

It should also be noted that the choice of crystalloids or colloids for fluid challenges is important – although our study initially showed comparable hemodynamic improvements within group 1 (Table 2). Because our trial assessed the initial hemodynamic effects of a distinct fluid bolus, it is unsurprising that hemodynamic effects of crystalloids and colloids were comparable in group 1. However, intraoperative longer-term effects were not observed. Considering the above issues, performing fluid boluses in a longer period than necessary may risk too liberal fluid administration and ineffective intravascular volume, potentially leading to additional adverse effects on patient hemodynamics. While a recent meta-analysis did not find any difference in severe postoperative complications and mortality between restrictive and liberal fluid approaches in major abdominal surgery, the incidence of major renal events was lower in liberal approach subgroups [19], a large cohort study found significant associations between high fluid volumes given during surgery with increased total costs resulting from prolonged hospital stays, and increased incidence of postoperative ileus for rectal and colon surgery patients [20].

The findings concerning increased noradrenaline requirements after prolonged fluid administration in group 2 provide further evidence that the rapid administration of a fluid challenge is accompanied by immediate intraoperative clinical benefits. This finding is also illustrated by the greater increase in SV and MAP after a rapid volume bolus in group 1. However, the overall amount of noradrenaline was comparable between the two treatment groups. Interestingly, reduced noradrenaline requirements and improved hemodynamic measurements were not observed when only crystalloid infusions were used. Moreover, the clinical efficacy of crystalloid infusions for altering intraoperative hemodynamic parameters was independent of the treatment group (Table 3). Only colloids caused significant differences in noradrenaline requirements, SV and MAP highlighting their well-known higher efficacy in restoring cardiac output in patients with clinical hypovolemia [21]. While colloids can cause adverse effects such as anaphylactic reactions, dilution coagulopathy, and endothelial barrier competence impairment, there is no evidence that they worsen perioperative outcomes [3, 22, 23]. Pathophysiologic considerations favor using colloids to restore the patient’s intravascular volume following perioperative blood losses during surgery in most GDT protocols [7].

Of course, several limitations of our trial must be acknowledged. Firstly, the limited initial sample size was further reduced because four (group 1) and five (group 2) patients did not require any fluid challenge during surgery, and thus, had to be excluded. Thus, for example, IOH duration and severity were numerically higher in group 2 without reaching statistical significance with the limited study size. Secondly, baseline crystalloid infusion (4 ml kg^−1^ h^−1^) was potentially set higher than necessary for restrictive fluid maintenance therapy during major spinal neurosurgery resulting in fewer requirements for a fluid bolus [13]. Thirdly, our study did not include critical ill patients limiting the transferability of the results to such patients due to significantly different pathophysiologic processes. Fourthly, patients’ intraoperative hemodynamic parameters were assessed during prone positioning and might be affected by decreased venous return and higher intrathoracic pressure. Because PPV is known to increase during prone positioning, PPV threshold was set higher in our study compared to other GDT protocols [8–11, 24]. We did not use the thermodilution method, which is the reference method for SV measurement. Therefore, the increase in SV induced by a fluid challenge may have been underestimated in our trial. Finally, hemodynamic measurements may be influenced by changes in surgical conditions or anesthesia depth while a fluid bolus was given impeding comparable conditions at the considered time points. This limitation may be particularly relevant to group 2 due to the longer period studied.

## Conclusions

In conclusion, evidence has been gained that a fluid bolus given within 5 min is more effective than fluid challenges given within 20 min. Therefore, a fluid challenge with high infusion rate should be the primarily treatment choice. Bolus infusions given within 20 min may result in volume administration without achieving relevant hemodynamic improvements, triggering adverse side effects. These findings should be accounted in future GDT protocols by specifying the time sequence in which a fluid bolus should be given.

## Supplementary Information

**Additional file 1: Supplementary Table 1.** Comparison of hemodynamics and catecholamine requirements between crystalloid and colloid infusion within 5 minutes.

**Additional file 2: Supplementary Table 2.** Comparison of hemodynamics and catecholamine requirements between crystalloid and colloid infusion within 20 minutes.

## Data Availability

The datasets used and/or analysed during the current study are available from the corresponding author on reasonable request.

## Abbreviations

AUC: Area under the curve
ASA: American Society of Anesthesiology
BMI: Body mass index
*CI*: Cardiac index
GDT: Goal directed fluid
IBW: Ideal body weight
IOH: Intraoperative hypotension
IQR: Interquartile range
KDIGO: Kidney Disease: Improving Global Outcomes
MAP: Mean arterial pressure
PPV: Pulse pressure variation
SV: Stroke volume
SVV: Stroke volume variation
