# Supplementary material for: Impact of the fluid challenge infusion rate on cardiac stroke volume during major spinal neurosurgery: a prospective single center randomized interventional trial
Source: BMC Anesthesiol. 2022 Dec 23;22:400. doi: 10.1186/s12871-022-01945-6 (PMC9783430; doi:10.1186/s12871-022-01945-6)
Supplement: Supplementary file 1 — Additional file 1: Supplementary Table 1. Comparison of hemodynamics and catecholamine requirements between crystalloid and colloid infusion within 5 minutes. [file 12871_2022_1945_MOESM1_ESM.docx]

**Supplementary Table 1.** Comparison of hemodynamics and catecholamine requirements between crystalloid and colloid infusion within 5 minutes.

| **Characteristics** | **Crystalloids n=29** | **Colloids n=41** | ***P*** |
| --- | --- | --- | --- |
| Before the fluid bolus | | | |
| Cardiac index [IQR] - l min^-1^ m-^2^ | 2.2 [1.7 - 2.6] | 2.0 [1.6 - 2.7] | 0.571 |
| Stroke volume [IQR] - ml | 67 [53 - 77] | 62 [53 - 72] | 0.442 |
| Mean arterial pressure [IQR] - mmHg | 74 [70 - 80] | 73 [67 - 79] | 0.650 |
| After fluid bolus | | | |
| Cardiac index [IQR] - l min^-1^ m-^2^ | 2.3 [1.8 - 2.7] | 2.2 [1.8 - 2.7] | 0.938 |
| Stroke volume [IQR] - ml | 74 [55 - 80] | 70 [57 - 80] | 0.858 |
| Mean arterial pressure [IQR] - mmHg | 80 [73 - 87] | 88 [78 - 94] | **0.031** |
| Alteration | | | |
| ΔSV [IQR] - ml | 2 [-2 - 9] | 5 [-1 - 9] | 0.339 |
| ΔSV [IQR] >10% - no (%) | 9 (31.0) | 19 (46.3) | 0.298 |
| ΔMAP [IQR] - mmHg | 4 [0 - 10] | 9 [6 - 16] | **0.017** |
| Catecholamines | | | |
| less - no (%) | 1 (3.5) | 6 (17.1) | 0.226 |
| equal - no (%) | 25 (86.2) | 33 (80.5) | 0.749 |
| more - no (%) | 3 (10.3) | 2 (4.8) | 0.642 |
| SV, stroke volume; MAP, mean arterial pressure. | | | |
