# Supplementary material for: Impact of the fluid challenge infusion rate on cardiac stroke volume during major spinal neurosurgery: a prospective single center randomized interventional trial
Source: BMC Anesthesiol. 2022 Dec 23;22:400. doi: 10.1186/s12871-022-01945-6 (PMC9783430; doi:10.1186/s12871-022-01945-6)
Supplement: Supplementary file 2 — Additional file 2: Supplementary Table 2. Comparison of hemodynamics and catecholamine requirements between crystalloid and colloid infusion within 20 minutes. [file 12871_2022_1945_MOESM2_ESM.docx]

**Supplementary Table 2.** Comparison of hemodynamics and catecholamine requirements between crystalloid and colloid infusion within 20 minutes.

| **Characteristics** | **Crystalloids n=26** | **Colloids n=23** | ***P*** |
| --- | --- | --- | --- |
| Before the fluid bolus | | | |
| Cardiac index [IQR] - l min^-1^ m-^2^ | 2.1 [1.8 - 2.7] | 2.3 [1.7 - 2.8] | 0.502 |
| Stroke volume [IQR] - ml | 61 [57 - 71] | 60 [58 - 70] | 0.984 |
| Mean arterial pressure [IQR] - mmHg | 75 [69 - 84] | 73 [68 - 78] | 0.554 |
| After fluid bolus | | | |
| Cardiac index [IQR] - l min^-1^ m-^2^ | 2.3 [1.9 - 2.5] | 2.5 [1.8 - 2.8] | 0.547 |
| Stroke volume [IQR] - ml | 63 [60 - 74] | 64 [60 - 68] | 0.880 |
| Mean arterial pressure [IQR] - mmHg | 78 [73 - 88] | 77 [71 - 87] | 0.873 |
| Alteration | | | |
| ΔSV [IQR] - ml | 1.5 [-4 - 7] | 2 [-2 - 7] | 0.794 |
| ΔSV [IQR] >10% - no (%) | 6 (23.1) | 6 (26.1) | 1.0 |
| ΔMAP [IQR] - mmHg | 0 [-6 - 11] | 3 [-1 - 10] | 0.366 |
| Catecholamines | | | |
| less - no (%) | 3 (11.5) | 5 (21.7) | 0.448 |
| equal - no (%) | 18 (69.2) | 12 (52.2) | 0.353 |
| more - no (%) | 5 (19.2) | 6 (26.1) | 0.817 |
| SV, stroke volume; MAP, mean arterial pressure. | | | |
